# Intraoperative reliability of the tibial anteroposterior axis “Akagi's Line” in total knee arthroplasty

**DOI:** 10.1002/jeo2.12020

**Published:** 2024-04-12

**Authors:** Kohei Kawaguchi, Ryota Yamagami, Kono Kenichi, Tomofumi Kage, Ryo Murakami, Takahiro Arakawa, Hiroshi Inui, Shuji Taketomi, Sakae Tanaka

**Affiliations:** ^1^ Department of Orthopaedics Tokyo University Hospital Bunkyo Tokyo Japan; ^2^ Present address: Department of Orthopaedics Tokyo University Hospital 7‐3‐1 Hongo Bunkyo 111‐0033 Tokyo Japan

**Keywords:** Akagi's line, anteroposterior axis, clinical outcome, functional outcome, navigation system, pain, rotational outlier, total knee arthroplasty

## Abstract

**Purpose:**

The tibial anatomical anteroposterior (AP) axis “Akagi's line” was originally defined on computed tomography (CT) in total knee arthroplasty (TKA); however, its intraoperative reproducibility remains unknown. This study aimed to evaluate the intraoperative reproducibility of the Akagi's line and its effect on postoperative clinical outcomes.

**Methods:**

This prospective study included 171 TKAs. The rotational angle of the intraoperative Akagi's line relative to the original Akagi's line (RAA) defined on CT was measured. The RAA was calculated based on the tibial component rotational angles relative to the intraoperative Akagi's line measured using the navigation system and CT. The effects of RAA on postoperative clinical outcomes and rotational alignments of components were also evaluated.

**Results:**

The mean absolute RAA (standard deviation) value was 5.5° (3.9°). The range of RAA was 22° internal rotation to 16° external rotation. Intraoperative Akagi's line outliers (RAA > 10°) were observed in 14% of the knees (24 knees). In outlier analysis, the tibial component rotation angle was externally rotated 6.5° (5.6°) in the outlier group and externally rotated 3.7° (4.2°) in the nonoutlier group (≤10°), with a significant difference between the two groups. Additionally, the outlier group (RAA > 10°) showed lower postoperative clinical outcomes.

**Conclusion:**

The original Akagi's line defined on CT showed insufficient reproducibility intraoperatively. The poor intraoperative detection of Akagi's line could be the reason for the tibial component rotational error and worse postoperative clinical outcomes.

**Level of Evidence:**

Level IV, case series.

**Abbreviations**
AP: anteroposterior
BCS: bicruciate‐substitute
CT: computed tomography
ICC: intraclass correlation coefficients
KOOS: Knee Injury and Osteoarthritis Outcome Score
KSS: 2011 Knee Society Scoring system
PCL: posterior cruciate ligament
RAA: The rotational angle of the intraoperative Akagi's line relative to the original Akagi's line
ROMA: range of motion and anatomical
SEA: surgical epicondylar axis
TKA: total knee arthroplasty

## INTRODUCTION

The rotational alignment of the tibial component is an important factor for successful total knee arthroplasty (TKA), and many surgeons usually determine the rotational angle of the tibial component based on the tibial anteroposterior (AP) axis. The Akagi's line [2] has been reported as one of the most reliable tibial AP axes [1, 8, 11, 12, 15, 20]. The Akagi's line was originally defined as the line that connects the middle of the posterior cruciate ligament (PCL) to the medial border of the patellar tendon (PT) attachment on computed tomography (CT) [2]. In a previous systematic review, the lowest errors or discrepancies from the projected transepicondylar axis were reported for the original Akagi's line among other tibial AP axes [12, 21]. Therefore, Akagi's line is considered the gold standard axis among tibial AP axes and a reliable landmark of the rotational alignment of the tibial component.

Nevertheless, many surgeons have adopted the reliable Akagi's line as an AP reference axis when determining the rotational angle of the tibial component intraoperatively. The accuracy of the rotational position of the tibial component is low [11]. The intraoperative inaccuracy of the Akagi's line could be a reason for the low tibial rotational accuracy [5, 23]. However, no study has evaluated the intraoperative reproducibility of the Akagi's line relative to the original Akagi's line. Thus, this study aimed to investigate whether the original Akagi's line can be reproduced intraoperatively using an intraoperative navigation system and CT evaluation software. The effect of the intraoperative reproducibility of Akagi's line on patient clinical outcomes after TKA was also assessed. We hypothesized that the intraoperative Akagi's line is an unreliable landmark and that its insufficient reproducibility could affect postoperative outcomes.

## MATERIALS AND METHODS

### Study design

This prospective study presents the results of 171 consecutive knees of 160 patients (133 women and 27 men; average age, 73.0 ± 7.7 years; average body mass index, 26.6 ± 4.3 kg/m^2^) who were diagnosed with osteoarthritis and varus deformity and underwent primary bicruciate‐substitute (BCS) TKA (JOURNEY II BCS; Smith & Nephew) using an image‐free computer navigation system (Precision N; Stryker Orthopedics) between May 2018 and June 2021. A total of 377 TKAs were performed during this period. To investigate the knee extension rotational angle, we excluded knees with a flexion contracture of >20° and those with a postoperative knee extension angle change of >10° relative to the preoperative angle (29 knees). We also excluded knees with valgus deformity (15 knees) and knees that underwent bicruciate‐retaining TKA (40 knees), TKA with other components (87 knees), revision TKA (8 knees), and TKA without a navigation system (8 knees) or perioperative CT (19 knees). Furthermore, whole‐leg coronal plane radiographs were acquired under weight‐bearing conditions to measure dynamic coronal limb alignment as a preoperative coronal hip–knee–ankle (HKA) angle. The surgeries were performed by four experienced knee surgeons. This study was approved by the institutional review boards of The University of Tokyo (No. 2674). The patients and their families were informed that the patient data will be submitted for publication, and written informed consent was obtained from them.

### Surgical procedure

All surgeries were performed using the soft‐tissue preserving method as described previously [6, 7, 8, 9, 25]. After registration of the navigation system, a 9.5 mm section of the lateral distal femur was sliced using the navigation system, and femoral alignment was maintained at 90° to the mechanical axis in the frontal plane and 4° flexion in the sagittal plane. For the proximal tibia, alignment was maintained at 90° to the mechanical axis in the frontal plane and 3° posterior slope in the sagittal plane. The femoral rotational axis was set parallel to the surgical epicondylar axis (SEA), and the tibial components were set according to the range of motion and anatomical (ROM‐A) technique, as reported previously [9]. This ROM‐A technique comprised two steps. First, the tibial component rotation was temporally set using the ROM technique. Second, the tibial component rotation was modified within the range between the intraoperative Akagi's line and the medial one‐third of the patella tendon. The patella was always resurfaced, and all components were cemented.

### Intraoperative reproducibility of the original CT‐defined Akagi's line

**Figure 1:**
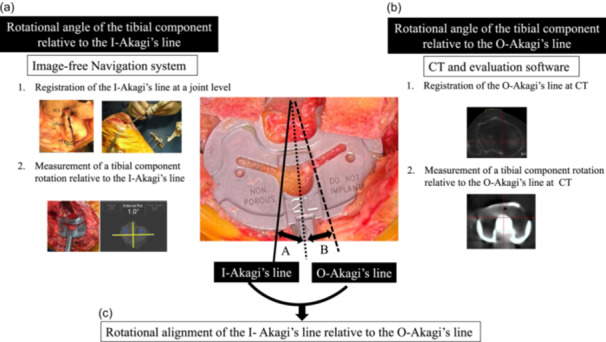
Scheme of our measurement method of the angle of the intraoperative Akagi's line and the original Akagi's line (RAA). (a) Measurement of the rotational angle of the tibial component relative to the intraoperative Akagi's line using the Image‐free navigation system. (b) Measurement of the rotational angle of the tibial component relative to the original Akagi's line using preoperative and postoperative CTs with the 3D software. (c) Measurement of the rotational alignment of the Intraoperative Akagi's line relative to the original Akagi's line from calculating angle A and angle B. CT, computed tomography; I‐Akagi's line, intraoperative Akagi's line; O‐Akagi's line, Original Akagi's line; PCL, posterior cruciate ligament; PT, patellar tendon.

To investigate whether the original CT‐defined Akagi's line could be reproduced intraoperatively, we measured the angle between the intraoperative Akagi's line and original Akagi's line (Figure 1). The rotational angle between the two Akagi's lines could not be measured intraoperatively directly because we used an image‐free navigation system, not a CT‐based navigation system. Therefore, the rotational angle between the two Akagi's lines was calculated by measuring the rotational angle of the tibial component relative to the two Akagi's lines. First, after surgical exposure, the Akagi's line [2] connecting the middle of the PCL to the medial border of the PT attachment at the tibial joint surface was registered on the navigation system. The final rotational angle of the tibial component was recorded relative to the intraoperative Akagi's line using the navigation system (Figure 1). Second, the original Akagi's line was defined using perioperative CT and 3D CT evaluation software Zed Knee System (LEXI). Further, the alignment of the components and other parameters were evaluated using a previously validated method described by Ueyama et al. (Figure 1) [16, 24, 26]. CT was routinely performed both before surgery and 2 weeks after surgery. Finally, the rotational angle of the intraoperative Akagi's line was measured relative to the original Akagi's line (RAA) using the rotational angles of the tibial component relative to the two Akagi's lines (Figure 1).

**Figure 2:**
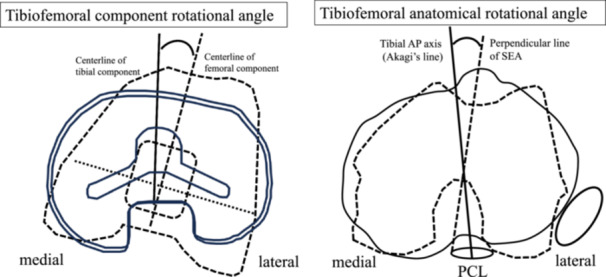
Tibiofemoral rotational alignment. AP axis, anteroposterior axis; SEA, surgical epicondylar axis.

The postoperative and preoperative angles of the Akagi's line relative to the line perpendicular to the femoral SEA on the tibial axial plane were also measured as the anatomical femorotibial rotational angle. The postoperative rotational angles of the tibial and femoral components were measured to determine the rotational angle of the tibiofemoral component (Figure 2).

### Postoperative clinical outcomes

Postoperative clinical outcomes were evaluated using the 2011 Knee Society Scoring system (KSS) [18, 22] and the Knee Injury and Osteoarthritis Outcome Score (KOOS) [17] 1 year postoperatively. The knee ROM was measured simultaneously and preoperatively using a standard clinical goniometer. We evaluated four separate categories in the 2011 KSS (symptoms, satisfaction, expectations and activity) and five separate categories in the KOOS (pain, symptoms, activities of daily living, sports, and quality of life).

### Outlier analysis to determine the reliability of the intraoperative Akagi's line

Regarding the intraoperative reproducibility of the original Akagi's line, an RAA value of >10° was defined as an outlier [24]. All knees were divided into the outlier (RAA > 10°) and nonoutlier (RAA within 10°) groups. Preoperative demographic and radiographic data were compared between the two groups to investigate the factors that affect the reproducibility of the Akagi's line. In addition, to investigate the effect of the intraoperative reliability of the Akagi's line on postoperative alignments and clinical outcomes, we compared the anatomical and component alignments as well as postoperative clinical outcomes between the two groups.

### Statistical analysis

All statistical analyses were performed using IBM SPSS Statistics v. 25.0 (IBM Corp.). Nonpaired t‐test and chi‐squared test were used for outlier analysis. All significance tests were two‐tailed, and a significance level of p < 0.05 was used for all tests. Statistical power analysis was performed after the study, which revealed α powers of 0.48 and 0.49 for the KOOS pain subscale scores and NKSS objective knee scores, respectively. The intraclass correlation coefficients (ICC) of the component rotational angles were evaluated using 3D CT evaluation software, and both values were found to be >0.70, indicating good reliability.

## RESULTS

**Figure 3:**
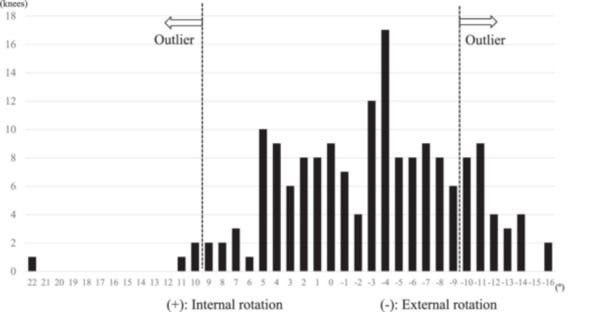
Distribution of the angle of the intraoperative Akagi's line relative to the original Akagi's line. An outlier was defined as the rotational angle of the intraoperative Akagi's line relative to the original Akagi's line of >10°.

The preoperative demographic data of the patients are shown in Table 1. The absolute value of RAA ranged from 0° to 22°, and the mean absolute value (standard deviation) was 5.5° (3.9°). The RAA was 2.9° (6.2°) externally rotated, and it ranged from internally rotated 22° to externally rotated 16°. The outlier of RAA (RAA > 10°) was detected in 14% of the knees (24/171 knees) (Figure 3). Table 2 presents the comparative analysis of the perioperative sociodemographic data of the outlier and nonoutlier groups. Significant differences were noted in the average preoperative knee extension angle (*p* < 0.01) and laterality (*p* = 0.04) between the two groups. In the outlier and nonoutlier groups, the rotational angle of the tibial component was 6.5° (5.6°) externally rotated and 3.7° (4.2°) externally rotated, and the anatomical tibiofemoral rotational angle (tibial internal rotation relative to the femur, +) was 7.0° (5.2°) and 4.3° (5.2°), respectively, showing significant differences (*p* = 0.02, *p* = 0.02) (Table 3). In addition, the KOOS pain subscale scores were 83.6 (12.1) and 88.3 (11.0), and the 2011 KSS symptoms subscale scores were 18.4 (4.5) and 20.2 (3.9) in the outlier and nonoutlier groups, showing significant differences between two groups (*p* = 0.04, *p* = 0.04) (Table 4).

**Table 1:** Preoperative demographic data.

	171 knees (160 patients)
Age, years	73.0 ± 7.7
Female: Male	133: 27
Right: Left	84: 87
Weight, kg	63.4 ± 12.7
Height, cm	154.4 ± 7.8
Body mass index, kg/m^2^	26.6 ± 4.3
Preoperative knee extension angle, degree	−8.3 ± 6.6
Preoperative knee flexion angle, degree	118.0 ± 14.8
Preoperative hip knee ankle angle, degree	170.2 ± 5.8
Preoperative tibiofemoral anatomical rotational angle (Internal rotation of tibia relative to femur: +)	1.2 ± 4.7

*Note*: mean ± standard deviation.

**Table 2:** Comparative analysis between the outlier and the nonoutlier group in perioperative demographic data.

	Outlier group	Nonoutlier group	*p* Value
Number, knees	24	147	‐
Age, years	73.8 ± 6.0	72.9 ± 7.9	n.s.
Right: Left	7: 17	77: 70	0.04*
Wight, kg	61.2 ± 12.1	63.8 ± 12.8	n.s.
Height, cm	154.5 ± 6.8	153.8 ± 7.9	n.s.
Body bass index, kg/m^2^	25.3 ± 4.6	26.9 ± 4.5	n.s.
Preoperative hip knee ankle angle, degree	170.9 ± 5.8	170.1 ± 5.9	n.s.
Preoperative knee extension angle, degree	−5.1 ± 6.8	−8.8 ± 6.4	<0.01*
Preoperative knee flexion angle, degree	119.6 ± 16.6	117.8 ± 14.5	n.s.
Postoperative knee extension angle, degree	−0.5 ± 2.1	−0.8 ± 2.1	n.s.
Postoperative knee flexion angle, degree	123.3 ± 9.8	122.2 ± 15.4	n.s.

*Note*: Outlier group (angle between the intraoperative Akagi's angle relative to the original Akagi's line more than 10°), significant difference.

Abbreviation: n.s., no significant difference.

* (*p* < 0.05), mean ± standard deviation.

**Table 3:** Comparative analysis between the outlier and the nonoutlier group in radiographic data.

	Outlier group	Nonoutlier group	*p* Value
Absolute value of rotational angle of intraoperative Akagi's line relative to original Akagi' line	12.2 ± 2.9	4.4 ± 2.8	<0.01*
Tibial component rotation angle (Internal rotation relative to original AP axis: +)	−6.5 ± 5.6	−3.7 ± 4.2	0.02*
Femoral component rotation angle (Internal rotation relative to SEA: +)	−1.4 ± 2.4	−0.7 ± 2.4	n.s.
Tibial component coronal angle (Varus alignment relative to mechanical axis: +)	0.1 ± 2.6	0.4 ± 1.2	n.s.
Femoral component coronal angle (Varus alignment relative to mechanical axis: +)	−0.4 ± 1.8	−0.5 ± 1.3	n.s.
Tibial component sagittal angle (Posterior inclination relative to mechanical axis: +)	2.3 ± 1.7	2.7 ± 1.6	n.s.
Femoral component sagittal angle (Flexion alignment relative to mechanical axis: +)	3.4 ± 3.5	3.3 ± 1.8	n.s.
Preoperative Tibiofemoral anatomical rotational angle (Internal rotation of tibia relative to femur: +)	2.3 ± 4.6	1.1 ± 4.7	n.s.
Postoperative tibiofemoral anatomical rotational angle (Internal rotation of tibia relative to femur: +)	7.0 ± 5.2	4.3 ± 5.2	0.02*
Postoperative tibiofemoral component rotational angle (Internal rotation of tibia relative to femur: +)	1.9 ± 7.7	1.4 ± 3.9	n.s.

*Note*: Outlier group (angle between the intraoperative Akagi's angle relative to the original Akagi's line more than 10°), significant difference.

Abbreviation: n.s., no significant difference.

* (*p* < 0.05), mean ± standard deviation.

**Table 4:** Comparative analysis between the outlier and the nonoutlier group in postoperative clinical outcomes.

	Outlier group	Nonoutlier group	*p* Value
Postoperative KOOS			
Pain	83.6 ± 12.1	88.3 ± 11.0	0.04*
Symptom	85.7 ± 11.3	85.1 ± 12.0	n.s.
ADL	85.6 ± 12.5	85.8 ± 12.6	n.s.
Sports	56.9 ± 26.4	54.1 ± 24.7	n.s.
QOL	68.5 ± 19.1	68.3 ± 21.1	n.s.
Postoperative 2011 Knee Society score			
Symptoms Score	18.4 ± 4.5	20.2 ± 3.9	0.04*
Satisfaction Score	28.3 ± 8.3	29.0 ± 8.2	n.s.
Expectation Score	9.9 ± 1.4	9.9 ± 2.4	n.s.
Functional Activity Score	72.9 ± 14.4	73.1 ± 19.8	n.s.

*Note*: Outlier group (Angle between the intraoperative Akagi's angle relative to the original Akagi's line more than 10°), significant difference.

Abbreviations: ADL, activity of daily living; KOOS, Knee injury and Osteoarthritis Outcome Score; n.s., no significant difference; QOL, Quality of life.

* (*p* < 0.05), mean ± standard deviation.

## DISCUSSION

This study revealed that the average angular difference between the intraoperative Akagi's line and original Akagi's line was 5.5° and that the outliers (RAA > 10°) were detected in 14% of the knees. The low reproducibility of the intraoperative Akagi's line led to poor rotational alignment of the tibial component and rotational mismatch in the anatomical tibiofemoral rotational alignment. In addition, the low reliability could lead to poorer postoperative clinical outcomes.

Regarding the reliability of the intraoperative Akagi's line relative to the original Akagi's line, Kuriyama et al. revealed that the intraoperative Akagi's line was highly variable and ranged from 18.6° of internal rotation to 14.7° of external rotation using CT‐based navigation [11]. This finding was consistent with that of the current study, showing a wide range of intraoperative Akagi's lines that were reproduced as the original Akagi's line. However, their study had a limitation in that their intraoperative study was performed on a tibial cutting surface based on other studies wherein the cutting surface was not appropriate to determine the intraoperative Akagi's line. This was because the PCL attachment was different on the tibial cutting surface compared with that on the tibial joint surface [8]. In this regard, the present study was more important than the previous one because we verified the intraoperative Akagi's line on the tibial joint surface. To overcome the limitation of the intraoperative low reliability of Akagi's line, we proposed two solutions: a CT‐based navigation system or robotics to determine the intraoperative Akagi's line and a mobile‐bearing TKA [10].

Our outlier analysis revealed that the intraoperative error of the Akagi's line occurred more frequently in the left knees and those with less flexion contracture. Regarding the laterality of knees, a study revealed that a tibial AP axis outlier (>5°) on the cutting surface relative to the AP axis on the joint surface occurred more frequently in the left knees [8]. Moreover, they argued that the likely reason was that all surgeons were right‐handed, and intraoperative handlings and views might influence their tibial rotations. In our cases, all surgeons were also right‐handed, which could be one of the reasons for laterality in the outlier analysis. Conversely, regarding knee flexion contracture, although a significant difference was observed between the outlier and nonoutlier groups, the difference was only 3°. This small difference could be clinically negligible. In addition, we determined the tibial AP axis at the 90° knee flexion position.

In this study, the outlier group showed more external rotation of the tibial component and higher tibiofemoral anatomical rotational mismatch than the nonoutlier group. No study has examined the relationship of the intraoperative reliability of the tibial AP axis with tibial component angle and tibiofemoral rotational angle mismatch. The external rotation of the tibial component in the outlier group could be attributed to the fact that the intraoperative Akagi's line was more externally rotationally defined than the original Akagi's line. In this study, malrotation of the tibial component could have induced the anatomical tibiofemoral rotational mismatch, as reported previously [24]. Both malrotation of the tibial component [3, 13, 19] and anatomical tibiofemoral rotational mismatch [17] were reported as risk factors for poor clinical outcomes. In this study, the KOOS pain scale score and 2011 KSS objective knee score in the outlier group were significantly lower than those in the nonoutlier group. These differences in the clinical outcomes could be related to malrotational alignments because other alignments were not different between the two groups. Therefore, the intraoperative identification of the Akagi's line may be important for the rotational alignment of the tibial component and tibiofemoral rotational mismatch as well as for postoperative clinical outcomes.

This study has several limitations. First, there might have been an error in identifying the original Akagi's line. We measured the Akagi's line using software based on both preoperative and postoperative CT. Previously, our interobserver ICC for the original Akagi's line was 0.75, which was moderate. In addition, Ueyama et al. showed high intraobserver and interobserver reliability for the same parameters analyzed in the current study [24]. Their intraobserver and interobserver ICCs of the original Akagi's line of the tibia were 0.93 and 0.92, respectively, consistent with our study. Second, the CT measurement procedures in the presence of flexion contracture were considered. Matsui et al. investigated the effect of contractures on the evaluation of rotational component alignment and revealed that the effects might be minimal if the flexion contracture was <15° [14]. Cases with flexion contracture of >20° on CT were excluded from this study. Consequently, the effect of contractures might be reduced. Third, this study was performed in patients who underwent BCS TKA only; therefore, the results may not apply to all TKA types. Further investigation is warranted to confirm whether this finding applies to conventional posterior‐stabilising or cruciate‐sustaining TKA components. Fourth, the difference in the KOOS pain subscale scores between the outlier and nonoutlier groups was below the minimum clinically relevant difference in the KOOS [4]. Fifth, the statistical power was only moderate; therefore, further research is warranted with larger sample sizes. In clinical, operators should take the intraoperative difficulty of the Akagi's line into consideration and should decide the rotation of the tibial component carefully.

## CONCLUSIONS

The original CT‐defined Akagi's line was not highly reproducible intraoperatively. The poor intraoperative detection of the Akagi's line could be the reason for the tibial component rotational error and worse postoperative clinical outcomes.

## AUTHOR CONTRIBUTIONS

**Kohei Kawaguchi** and **Hiroshi Inui**: Conceptualization, methodology. **Kohei Kawaguchi, Hiroshi Inui, Ryota Yamagami, Ryo Murakami, Tomofumi Kage, Takahiro Arakawa**, and **Kono Kenichi**: Surgy and Investigation. **Kohei Kawaguchi**: Writing—original draught preparation. **Kohei Kawaguchi, Ryota Yamagami, Shuji Taketomi**: Writing review and editing. **Sakae Tanaka**: Supervision.

## CONFLICT OF INTEREST STATEMENT

The authors declare no conflict of interest.

## ETHICS STATEMENT

The institutional review board at the Tokyo University hospital approved this retrospective study.

The patients and their families were informed that the patient data will be submitted for publication, and written informed consent was obtained from them.

## Data Availability

The data sets used and/or analysed during the current study are available from the corresponding author on reasonable request.
